# Alternans and Spiral Breakup in an Excitable Reaction-Diffusion System: A Simulation Study

**DOI:** 10.1155/2014/459675

**Published:** 2014-11-12

**Authors:** M. Osman Gani, Toshiyuki Ogawa

**Affiliations:** ^1^Meiji Institute for Advanced Study of Mathematical Sciences, Meiji University, 4-21-1 Nakano, Nakano-ku, Tokyo 164-8525, Japan; ^2^Graduate School of Advanced Mathematical Sciences, Meiji University, 4-21-1 Nakano, Nakano-ku, Tokyo 164-8525, Japan

## Abstract

The determination of the mechanisms of spiral breakup in excitable media is still an open problem for researchers. In the context of cardiac electrophysiological activities, spiral breakup exhibits complex spatiotemporal pattern known as ventricular fibrillation. The latter is the major cause of sudden cardiac deaths all over the world. In this paper, we numerically study the instability of periodic planar traveling wave solution in two dimensions. The emergence of stable spiral pattern is observed in the considered model. This pattern occurs when the heart is malfunctioning (i.e., ventricular tachycardia). We show that the spiral wave breakup is a consequence of the transverse instability of the planar traveling wave solutions. The alternans, that is, the oscillation of pulse widths, is observed in our simulation results. Moreover, we calculate the widths of spiral pulses numerically and observe that the stable spiral pattern bifurcates to an oscillatory wave pattern in a one-parameter family of solutions. The spiral breakup occurs far below the bifurcation when the maximum and the minimum excited states become more distinct, and hence the alternans becomes more pronounced.

## 1. Introduction

Cardiovascular diseases and sudden cardiac deaths are major issues nowadays facing researchers in the fields of medical sciences and mathematical physiology throughout the globe, especially in the industrialized world. Nearly fifteen million [1] individuals die every year in the universe because of cardiovascular diseases. Per annum, these diseases, in the United States alone, have nearly one million deaths, which is over 40% of all deaths [1]. A study [1] shows that more than 50% of sudden cardiac deaths happen in humans without any previous symptoms. The most common and comparatively less dangerous types of heart diseases are coronary heart disease, heart failure, heart stroke, heart attack, atrial fibrillation, and so forth. In this paper, we focus on cardiac arrhythmias such as ventricular tachycardia (VT) and ventricular fibrillation (VF). The most irregular, life threatening, and deadly case of cardiac arrhythmia is known as ventricular fibrillation, which occurs when the heart is not able to pump oxygen-rich blood to the body and results in death of body cells and heart cells [2].

During the last few decades, many research groups have been performing research in this area in order to understand the proper electrophysiological activity of cardiac tissues. Hodgkin and Huxley [3] proposed the first quantitative mathematical model, a four-variable (*V*, *m*, *n*, and *h*) model, in order to describe action potential propagation through very careful experimentation on squids (squid giant axon). They open a door for researchers in the area of mathematical physiology to model various nonlinear complex phenomena. Modifying Hodgkin and Huxley model in 1962, Noble established the first physiological model to identify the electrical activity of cardiac cells [4]. Several ionic models [5–12] have been developed to further understand the precise mechanism of electrical activity of cardiac cells. Ionic models accurately reproduce most of the underlying properties of cardiac cell dynamics. However, ionic models are not largely suitable for modeling many important properties of an excitable cardiac cell such as cardiac arrhythmias. Although some simplified ionic models [11–13] can help in the study of cardiac arrhythmias, they are not capable of reproducing the accurate shape of the action potentials.

For excitable media, researchers often employ a two-variable partial differential equation (PDE) model, which is known as FitzHugh-Nagumo (FHN) model [14, 15]. The FHN model is a simplified variant of the Hodgkin and Huxley model, which traces the fast-slow dynamics of excitable system of a spiking neuron. However, it is not suitable to explain the cardiac electrical activity. The simulation results of this model fail to give some qualitative properties of cardiac tissues such as the proper shape of action potentials of a cardiac cell and the restitution properties of tissues. Karma [16] showed that three essential properties of the Noble model [4], the wavefront insensitivity, phase-wave back and alternans, are absent in the FHN model. Aliev and Panfilov [17] proposed a simplified PDE model of cardiac tissue, which is capable of improving the shape of action potentials.

One of the characteristic features of excitable media is the spontaneous formation of spiral wave. The spiral wave pattern is the only pattern that survives in an excitable medium without any external stimulus [18]. This pattern is observed at the initial stage of a diseased heart. However, the most dangerous type of pattern is the spiral wave breakup. The spiral breakup behavior was first noticed in the calculation of a *λ*-*ω* system, in which the system was entirely different from the models of cardiac tissue [19]. After nearly a decade, this behavior was rediscovered in the several ionic models of cardiac tissue [20–23] and in cellular automata models of cardiac excitable media [24, 25]. Later on, several mechanisms of spiral breakup were established by researchers using ionic models [26, 27] and two-variable PDE models [16, 17, 28–35]. The concept of wave-break [36] is important in the mechanism of spiral breakup in a normal as well as diseased human heart tissue.

In two-dimensional reaction-diffusion PDE system, several mechanisms of spiral breakup were discovered by researchers. These include tissue size in a relatively homogeneous undamaged tissue [28], delayed-inhibitor production [29], lateral instability [30], and superexcitability in the threshold dynamics [31]. The alternans in action potential duration (APD) of sufficiently large amplitude can also break up an isolated rotating spiral wave [16]. It was also shown in [32] that spiral breakup occurs when the relative refractory period is shorter than the absolute refractory period. Panfilov in [33] showed that spiral waves can break up into a complicated spatiotemporal pattern simply due to the inherent dynamics of an excitable medium without heterogeneities. Panfilov and Zemlin showed that the breakup occurred if the slope of the restitution curve is steeper than −1 [34, 35]. However, researchers are still not quite clear about the exact mechanism of spiral breakup.

The objective of the present study is to analyze alternans and spiral breakup numerically within a proper choice of parameters and determine a dynamical mechanism of spiral breakup. In this respect, we propose a FHN-type reaction-diffusion system for excitable media. Firstly, we show the transverse instability of the planar traveling wave solution. Secondly, we observe spiral instability using the same set of parameters. The instability manifests as a function of a parameter *b* in the recovery equation. We characterize the instability numerically by considering the maximum and minimum lengths of the excited state. Finally, we show that unstructured spiral breakup, which leads to complex spatiotemporal patterns, is preceded by the front and the back interaction of excited states or alternans instability.

The rest of the paper is structured as follows. In Section 2, we present our two-variable PDE model for excitable media and computational methodology.  Section 3 presents numerical results and discussions in one and two dimensions. We investigate the stability of planar traveling wave and link the results to the complex dynamics of spiral waves. We show a close dynamical corresponding between spiral wave solution in two dimensions and the periodic traveling wave solution in one dimension. We conclude the paper in Section 4.

## 2. Mathematical Model and Methods of Computation

### 2.1. Mathematical Model

**Figure 1:**
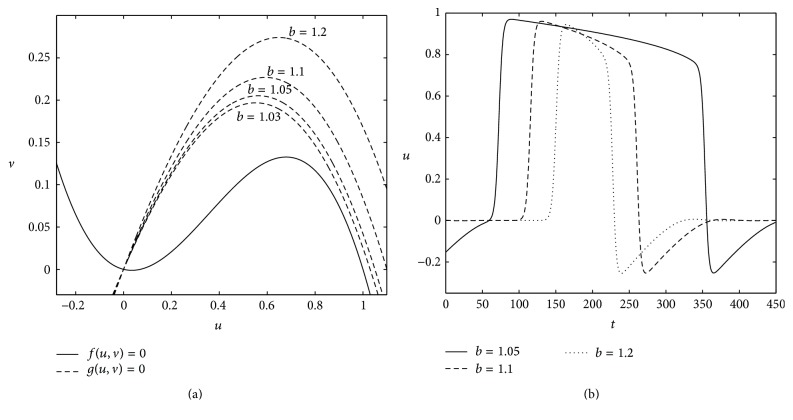
(a) The nullclines of model (1). Solid line is the *u*-nullcline and dashed lines are the *v*-nullcline for different values of the parameter *b*. (b) The action potential of (1) corresponding to the three different values of *b* in (a), that is, *b* = 1.2, 1.1, and 1.05. The parameter settings are the same in Table 1.

In this paper, we propose a reaction-diffusion system for excitable media to mimic cardiac electrical activities as reported in [37]. The model consists of two equations describing fast and slow dynamics of the system and it is given as follows:

(1) $$\begin{array}{c} \frac{\partial u}{\partial t}=d_{u}\Delta u+u\left(1-u\right)\left(u-a\right)-v,\\ \frac{\partial v}{\partial t}=d_{v}\Delta v+\epsilon \left(du\left(b-u\right)\left(u+c\right)-v\right), \end{array}$$

where the reaction terms *f*(*u*, *v*) = *u*(1 − *u*)(*u* − *a*) − *v* and *g*(*u*, *v*) = *ϵ*(*du*(*b* − *u*)(*u* + *c*) − *v*) describe the local kinetics of variables *u* and *v*. The small parameter *ϵ*, 0 < *ϵ* ≪ 1, describes the ratio of time scales of variables *u* and *v*. The fast activator variable *u* and the slow inhibitor variable *v* are known as the excitable and recovery variable, respectively. The variables *u* and *v* are also referred to as the propagator and controller variables, respectively. The variable *u* stands for the membrane potential and *v* stands for the conductance of the inward current in the context of cardiac electrical activities. The nullclines of (1) are plotted in Figure 1(a). The *u*-nullcline is *N*-shaped, which is similar to that of the standard FHN model [14, 15]. The *v*-nullcline is not linear or monotone like the FHN model. This type of *v*-nullcline for the dynamics of recovery variable is more appropriate for the cardiac electrical activities [17]. Both nullclines intersect each other at a point, which is called the rest state of the excitable media. The point corresponds to the (0,0) state as shown in Figure 1(a). Thus, there exists only one possible steady state solution. The parameter *a* is called the excitation threshold, which lies between 0 and 1/2, that is, 0 < *a* < 1/2. For a small perturbation of *u* less than the threshold value, that is, *u* < *a*, the system reverts to the rest state; otherwise (i.e., *u* > *a*) the system undergoes long excursions with fast-slow dynamics in the (*u*, *v*)-plane before reverting to the stationary (i.e., rest) state. Therefore, the system can be categorized as excitable according to [38]. Another parameter, *b*, plays a crucial role in model (1), by controlling the period of excitation of the medium. The two nullclines intersect each other again at the right knee, when *b* = 1. Thus, the parameter *b* can be assigned any value greater than 1. The excitation period becomes larger and larger when *b* approaches 1 but decreases when *b* increase (see Figure 1(b)). The parameter, *b*, is responsible for creating gaps between two nullclines at the right knee of the *uv*-plane (see Figure 1(a)). This is key to controlling the velocity of the solution at the right slow manifold. Therefore, the nonlinear kinetic of the slow variable *v* is responsible for the slow movement of solutions at the right knee of the *u*-nullcline, compared to the original FHN model. We have modified the FHN equations without changing the slow manifold. However, we changed the velocity on each branch of the slow manifold. This improves the shape of the action potential to mimic the shape of a real cardiac action potential (see Figure 1(b)). We also point out that the cardiac action potential is very different to that seen in neuron action potential. It has a prolonged plateau phase lasting around 300 milliseconds (ms) compared with the 1 ms seen in nerves [39]. Figure 1(b) shows action potential of (1) for three different values of *b*. The duration of the excited phase is referred to as the action potential duration (APD) (see Figure 1(b)) and the recovery period is referred to as the diastolic interval (DI), that is, the duration between two consecutive excited phases in a periodically stimulated cell. Figure 1(b) shows that the APD is increasing as *b* decreases. Since we used FHN kinetic for the fast variable, the recovery phase does not return to the rest state immediately but does so after a large hyperpolarization. Although this is sometimes seen in nerve cells, in cardiac action potential, the recovery phase returns to rest state without hyperpolarization. Apart from the excitation threshold parameter, a, the parameters of our model do not have clear physiological meanings like most of the other FHN-type models [16, 17]. In our case, the parameters are adjusted to reproduce some of the macroscopic characteristics of cardiac tissues such as the shape of the action potential, dispersion relation, refractoriness, and the restitution of APD [40].

### 2.2. Methods of Computation

For the numerical computations in two dimensions, we used the alternating-direction implicit (ADI) method with Neumann boundary conditions. In this method, the iteration is explicit in one direction and implicit in the other direction in the first half-timestep and in the second half-timestep the order is reversed. We sought for numerical solutions on the spatial grid (*x*_*i*_, *y*_*j*_) with *x*_*i*_ = *i*Δ*x*, *i* = 0,…, *N*_*x*_, and *y*_*j*_ = *j*Δ*y*, *j* = 0,…, *N*_*y*_, where Δ*x* = Δ*y* for a uniform mesh grid and time *t*_*n*_ = *n*Δ*t*, *n* = 0,1, 2,3,…, where Δ*t* is the time step. The space steps in the *x*-direction and in the *y*-direction are therefore defined, respectively, by

(2) $$\begin{array}{c} \Delta x=\frac{L_{x}}{N_{x}},\;\Delta y=\frac{L_{y}}{N_{y}},\;N_{x},N_{y}\in Z, \end{array}$$

where the size of the domain in the (*x*, *y*)-plane is 0 < *x* < *L*_*x*_ and 0 < *y* < *L*_*y*_. For the numerical approximation of the state variables, in (1), we denote the grid approximations by *U*_*i*,*j*_^*n*^ ≈ *u*(*x*_*i*_, *y*_*j*_, *t*_*n*_) and *V*_*i*,*j*_^*n*^ ≈ *v*(*x*_*i*_, *y*_*j*_, *t*_*n*_), such that the full discrete approximation of *U*_*i*,*j*_^*n*^ is given by

(3) Ui,jn+1/2−Ui,jnΔt/2=duUi−1,jn+1/2−2Ui,jn+1/2+Ui+1,jn+1/2Δx2+duUi,j−1n−2Ui,jn+Ui,j+1nΔy2+fUi,jn,Vi,jn,

(4) Ui,jn+1−Ui,jn+1/2Δt/2=duUi−1,jn+1/2−2Ui,jn+1/2+Ui+1,jn+1/2Δx2+duUi,j−1n+1−2Ui,jn+1+Ui,j+1n+1Δy2+fUi,jn+1/2,Vi,jn+1/2.

Equation (3) represents the first half-timestep and (4) represents the second half-timestep. The formula *δ*_*x*_ *U*_*i*,*j*_^*k*^ = *U*_*i*+1/2,*j*_^*k*^ − *U*_*i*−1/2,*j*_^*k*^ is the central difference operator, and a similar formula holds true for *δ*_*y*_. An equivalent discrete system of equations can also be written for *V*_*i*,*j*_^*n*^. For the full details of the method we refer to [41, 42]. The advantage of this method is that it is unconditionally stable and second order in time and space [43]. Nevertheless, there is still a stability condition for the convergence of the solutions [41]. For the numerical computation in spiral wave formation, in order to initiate the first spiral, we used a one-dimensional band of traveling wave as an initial guess. This corresponds to a two-dimensional broken wave front. The break is located at the middle of the medium. For the computations in one dimension, we used an implicit scheme with periodic boundary conditions on [0, *L*_*x*_]. In this study, we considered the parameter, *b*, as a free parameter while other parameter values are fixed according to Table 1, unless otherwise stated.

For the calculation of different spiral pulse widths (see Figure 6) we used Algorithm 1.

## 3. Numerical Results and Discussion

### 3.1. Planar Wave Instability

**Figure 2:**
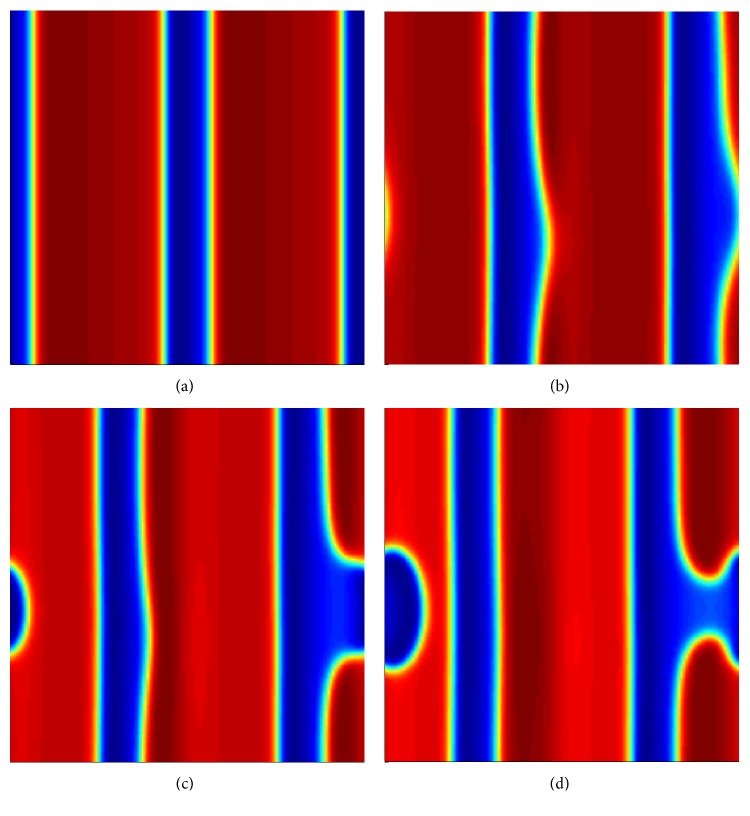
Breakup of planar pulses in model (1) by transverse instability. The snapshots are at time (a) *t* = 0, (b) *t* = 187.62, (c) *t* = 199.32, and (d) *t* = 220.12. Numerical integration with space step *dx* = *dy* = 0.2 and time step *dt* = 0.01 on the grid of 150 × 150 elements. The red area represents excited state and the blue area represents the resting state of the tissue.

Planar wave propagation in one direction is a simpler case of wave propagation compared to spiral wave propagation in a two-dimensional system. Wave propagation failure in excitable media very often leads to the beginning of spatiotemporal disorder. In the context of electrophysiological activities, it may lead to ventricular fibrillation [44]. In this subsection, we study the stability of periodic planar traveling wave solutions numerically. We used the ADI method, described in Section 2, with periodic boundary conditions on (0, *L*_*x*_)×(0, *L*_*y*_). The parameter settings are the same as those in Table 1. We observed that the failure of pulse propagation (wave breakup) of planar wave occurs when a small random perturbation is applied to the stable planar wave solution near the bifurcation point. We define the system size *L*_*x*_≔*n* × *l*, where *n* is the number of wave pulse and *l* is the spatial period. First, we consider a periodic stable planar wave train of two pulses for a system size of *L*_*x*_ = 30, propagating in the negative *x*-direction with constant velocity (see Figure 2(a)). Here, we are concerned with the stability of the periodic planar traveling wave of spatial period *l* = 15, so as to compare the results with the spiral wave instability with the same spatial period. The spatial period *l* = 15 is also the minimum stable spatial period near the onset of the instability of spiral waves. In the stable planar wave case (see Figure 2(a)), the value of *b* is 1.035.  Figure 2 displays the breakup of planar pulse by transverse instability. We introduced a suitable small random perturbation in the initial data and consider a range of values of *b*. In Figure 2, it can be seen that the initial stable planar wave becomes unstable as time develops. The behavior of the system at time *t* = 187.62 is clearly different from the observation in Figure 2(a), in which a dent is formed (see Figure 2(b)). With the development of time, the dent grows and its curvature becomes more pronounced (see Figure 2(c)). As a result, a movement of the wave in the transverse direction is induced into the system with the formation of spiral tips that protrude towards the center of the curvature (see Figure 2(d)). This behavior is usually followed by a domain breakup in the two-dimensional system. We refer this instability of planar wave as transverse instability [44]. The transverse wave instability was also found experimentally in an excitable Belousov-Zhabotinsky (BZ) reaction [45].

### 3.2. Spiral Breakup Phenomena

**Figure 3:**
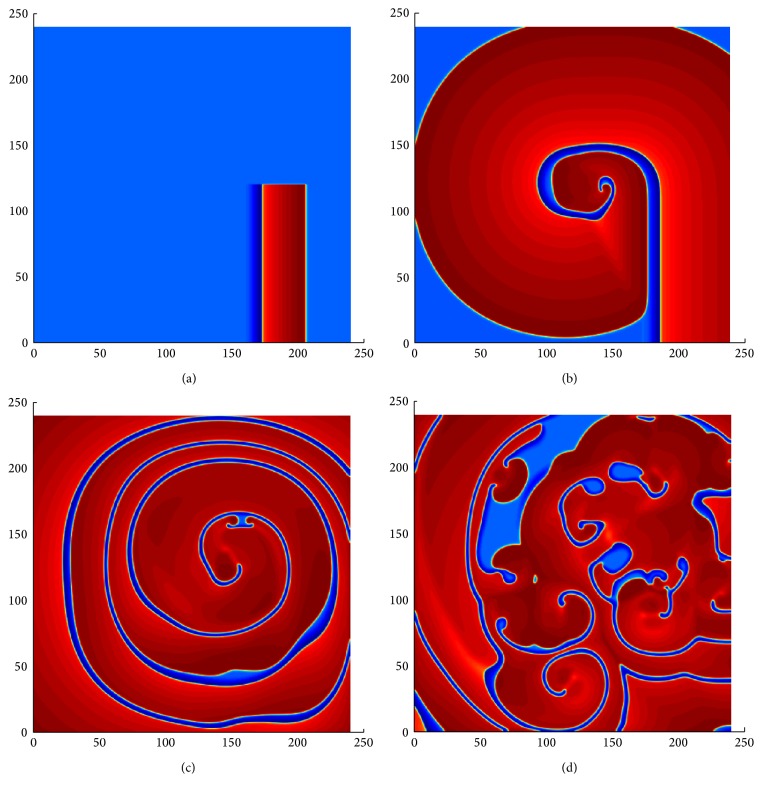
Time sequence illustrating the dynamical mechanism of spiral breakup in (1). The parameter settings are same as in Table 1 with *b* = 1.015. The pictures are at time (a) *t* = 0, (b) *t* = 546, (c) *t* = 2270, and (d) *t* = 4909. Numerical integration with space step *dx* = *dy* = 0.25 and time step *dt* = 0.05 on the grid of 960 × 960 elements. The red area represents excited state and the blue area represents the resting state of the tissue.

In this section, we investigate the dynamics of spiral wave solutions in two dimensions as a function of the parameter *b* for two different initial profiles. The parameter *b* controls the emergence of stable spiral wave patterns and spiral breakup in the two-dimensional simulation. As we mentioned earlier, the value of *b* is responsible for the creation of gaps between the two nullclines at the right knee of the *uv*-plane; that is, the propagation velocity of the solution is slower at the right slow manifold for a smaller value of *b*. As a result, the excited state becomes much larger (smaller) for smaller (larger) values of *b*. The first initial condition (see Figure 3(a)) we used here is a band of traveling wave. It was generated from the simulation result of a one-dimensional traveling wave data. The 2D broken wave front is located at the middle of the excitable tissue. Using this initial guess, we observed a beautiful spiral wave pattern (see Figure 5(a)) by using *b* = 1.05. The precise mechanism of spiral breakup, that is, ventricular fibrillation, is still not quite clear to researchers in the context of cardiac arrhythmias. In this paper, we showed that the spiral breakup occurs mainly because of the oscillation of spiral pulses and the interaction between the front and the back of the oscillatory wave pulses far away from the bifurcation. In many cases, the initial conditions are also important to determine the spiral wave breakup [26]. Thus, in order to understand the initiation and evolution of wave propagation more precisely in model (1), we considered two types of initial data. The first initial condition is given in Figure 3(a), as mentioned earlier. We used the ADI method, described in Section 2, with Neumann boundary conditions. The problem was considered numerically with *dt* = 0.05; *dx* = *dy* = 0.25 on a grid of 960 × 960 elements. The spatial domain length is *L*_*x*_ = *L*_*y*_ = 240. The parameter values used in the numerical simulation are given in Table 1.  Figure 3 shows the evolution of spatial patterns at *b* = 1.015. In this case, after several revolutions of the spiral wave (see Figure 3(b)), the initial breakup occurred in the vicinity of the core of the spiral (see Figure 3(c)). Gradually, the excited state covered most regions of the medium. Moreover, we also observe that when the rest region is limited, part of the excited region breaks and disappears, making the medium spatially disorganized. As a result, new spiral waves are produced to occupy the space created due to the vanishing spiral waves. That is, a new wave break produces two other new spirals. This process continues repeatedly throughout the medium and, at *t* = 4909, a chaotic phenomenon of waves appears in the form of a complicated spatial distribution of the propagator variable (see Figure 3(d)). Similar results were obtained with smaller space and time steps. This chaotic pattern in the heart is called ventricular fibrillation.

**Figure 4:**
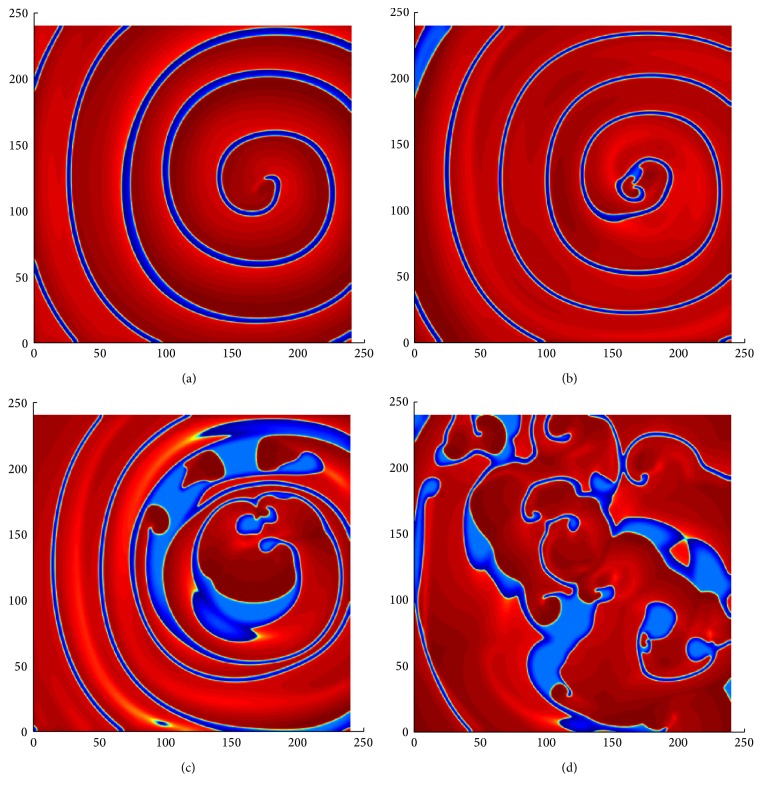
The spiral breakup in (1), when *b* = 1.015. The pictures are at time (a) *t* = 0, (b) *t* = 1266, (c) *t* = 2356, and (d) *t* = 3657. Numerical integration with space step *dx* = *dy* = 0.25 and time step *dt* = 0.05 on the grid of 960 × 960 elements. The red area represents excited state and the blue area represents the resting state of the tissue.

Another initial data was obtained for *b* = 1.025 (see Figure 4(a)) by the continuation from a stable spiral wave using different values of *b*, that is, considering the solution of one step as an initial guess for the next step, in such a way that the value of *b* is decreased in each subsequent step. Note that, it is difficult to prepare initial condition with stable spiral near the breakup. The parameter settings are similar as in Figure 3. We observed the first initial breakup at the core of the spiral wave at time *t* = 1266 (see Figure 4(b)). However, after the initiation of the spiral breakup, the medium spanned by the excited state enlarges (see Figure 4(c)) and further breakup is initiated in the medium away from the core. Eventually, this gives rise to the complicated phenomenon in Figure 4(d), at time *t* = 3657. Note that, in both cases, the process of breakup starts in the vicinity of the center of the initial spiral and spreads throughout the medium. However, the first major breakup is not similar in both cases (see Figures 3(c) and 4(b)). Thus, initial data are important for the process of a spiral breakup with a proper choice of parameters.

### 3.3. Spiral Wave Phenomena as a Function of the Parameter *b*

**Figure 5:**
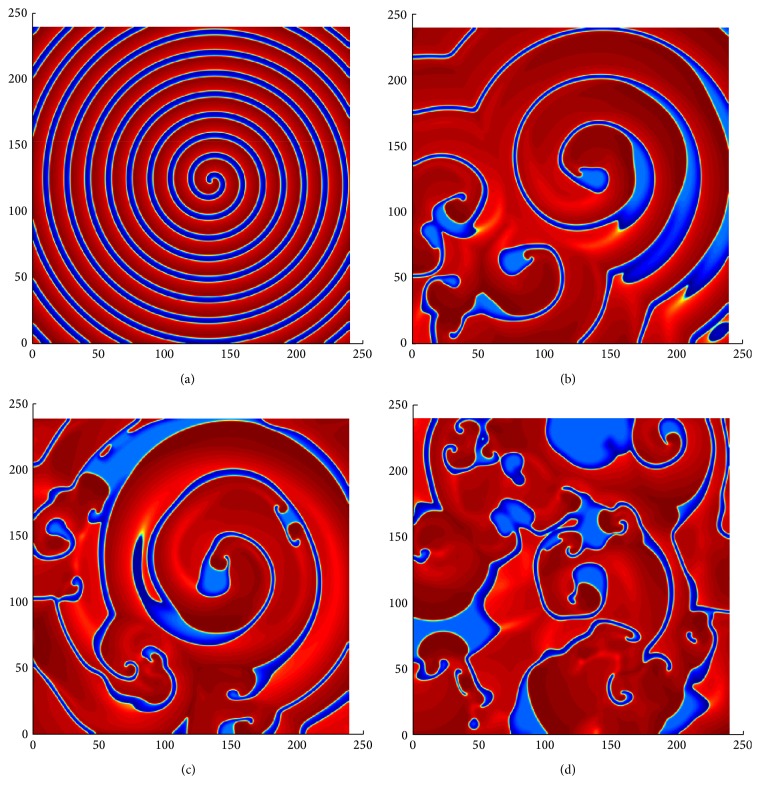
The spiral dynamics of the tissue as a function of parameter *b* in (1). The panels are at (a) *b* = 1.05, (b) *b* = 1.02, (c) *b* = 1.018, (d) *b* = 1.015. Numerical integration with space step *dx* = *dy* = 0.25 and time step *dt* = 0.05 on the grid of 960 × 960 elements.

Figure 5 illustrates the spiral wave behavior as a function of the parameter *b*. In this model, decreasing the value of *b* increases the widths of the spiral pulses, that is, the length of the excited state. The parameter values are given in Table 1. The first panel of Figure 5 shows a stable spiral wave pattern for *b* = 1.05. The stable spiral wave becomes progressively destabilized by increasing oscillation in wavelength (distance between two consecutive fronts or back) along the spiral wave arm [46] (i.e., a decrease at *b* = 1.02) and breakup occurs without affecting the two inner spiral rotations (see Figure 5(b)). Panel (c) of Figure 5 shows the breakup closer to the core, with only a single spiral remaining unbroken, as *b* is further increased (*b* = 1.018). Finally, with an additional increase in *b*, (*b* = 1.015), no further complete rotation of the spiral exists and the breakup spreads across the entire medium (see Figure 5(d)). The breakup repeats itself until the chaotic pattern is fully developed. The same behavior was observed in the two-variable Karma model [28]. Therefore, in the model (1), we considered *b* as a bifurcation parameter, which is capable of showing the wave pattern from tachycardia to fibrillation.

### 3.4. Dynamical Behavior of Spiral Wave Solutions and the Corresponding Periodic Traveling Wave Solutions in 1D

#### 3.4.1. Calculation of Widths of Spiral Pulses

**Figure 6:**
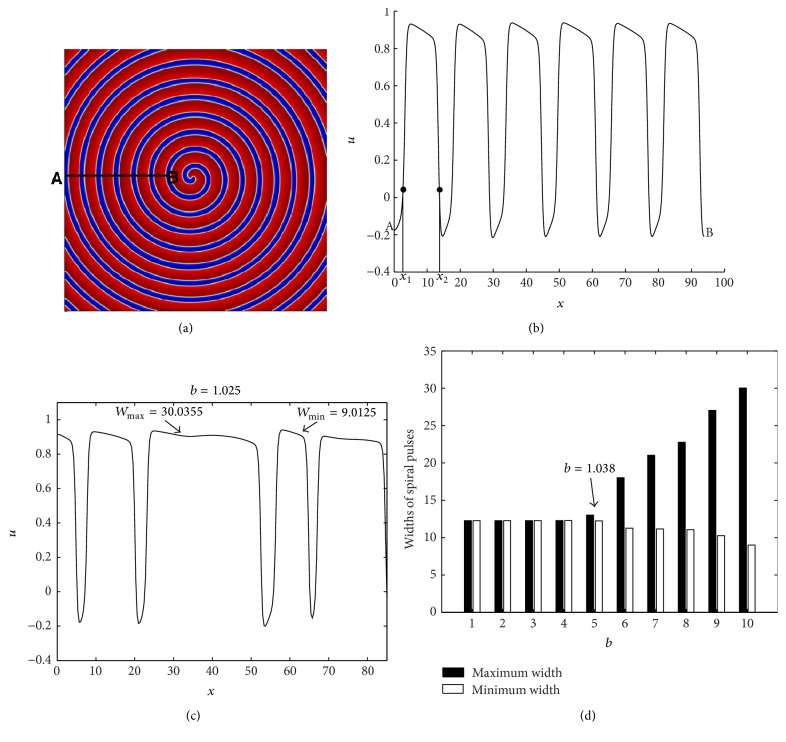
(a) The two-dimensional plot *u*(*x*) for *b* = 1.05 of (1). (b) The line AB in (a) gives a one-dimensional plot of *u*(*x*) by two-dimensional interpolation. (c) A one-dimensional cross-sectional plot of *u*(*x*), when *b* = 1.025. (d) The maximum and minimum widths of spiral pulses as a function of the parameter *b* of (1). The values of *b* on the *x*-axis are 1.06, 1.05, 1.045, 1.04, 1.038, 1.037, 1.035, 1.032, 1.03, and 1.025 (from left to right).

We developed an algorithm (see Algorithm 1) that calculates the widths of different spiral pulses numerically. The widths are calculated by a transverse interpolation of the 2D spiral wave along a line AB (see Figure 6(a)). The interpolation was done in such a way that the tip (or core) of the spiral wave is excluded. This consideration makes sense since the spiral wave is symmetric on either side of the core. For each pulse of the 1D interpolated data of *u*, we determined two points (see, e.g., Figure 6(b)); one is at the front and the other is at the back of the pulse. The location of the points lies near the zero (positive side) of the function *u*. The strategy employed here is simply based on the behavior of the cross-section of the spiral wave, which alternates between a negative value and a positive value within each pulse. The distance between the two points as measured on the *x*-axis gives the pulse width for that pulse, that is, the pulse width is defined as *W* = |*x*_1_ − *x*_2_| (see Figure 6(b)). For example, in Figure 6(b), the width of the first pulse is |*x*_1_ − *x*_2_ | = 12.2628. This process is repeated for all the pulses along AB. We are concerned in determining the maximum and minimum widths as a function of *b*, in order to find an oscillatory pattern of solutions.  Figure 6(c) shows the oscillation of pulse widths when *b* = 1.025. At this stage Algorithm 1 gives *W*_max⁡_ = 30.0355 and *W*_min⁡_ = 9.0125.  Figure 6(d) shows the maximum and the minimum widths of spiral pulses as a function of the parameter *b*. We started the calculation for a stable spiral pattern (i.e., equal widths of spiral pulses) at *b* = 1.06, while other parameters are kept fixed according to Table 1. In Figure 6(d), the following consecutive values of parameter *b* are considered: *b* = 1.06, 1.05, 1.045, 1.04, 1.038, 1.037, 1.035, 1.032, 1.03, and 1.025 in each calculation (from left to right). This means that, in each computation, the value of *b* is gradually decreased until the onset of oscillation of the pulse width (i.e., alternans) occurs. The oscillation is initiated at a value of *b* = 1.038. This shows that, in our numerical results, the stable spiral wave pattern bifurcates to an oscillatory wave pattern at about *b* = 1.038. Subsequently, the oscillations increase for decreasing the values of *b*. These oscillations become more intense when the value of *b* is far below the onset of the instability. Note that the width of the spiral pulses increases, allowing the number of pulses formed to decrease.

#### 3.4.2. Alternans in Spiral Pulse Solution

**Figure 7:**
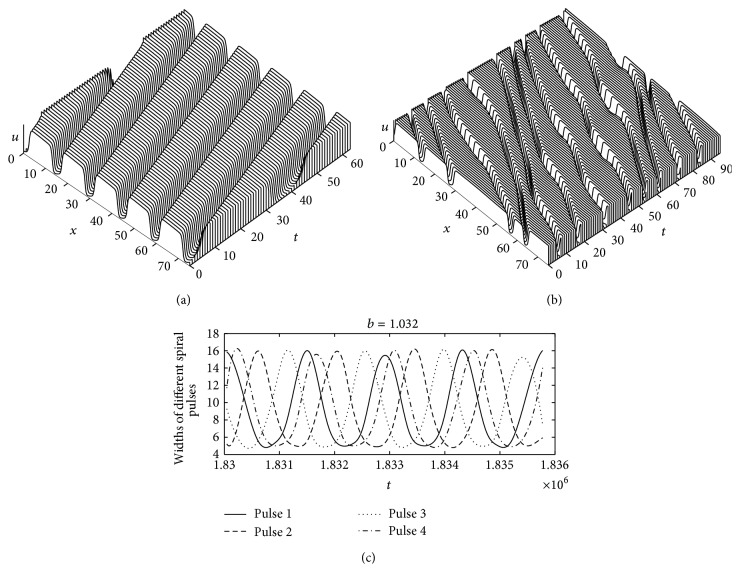
Time sequence illustrating the dynamics of spiral waves in two dimensions. The plots in (a) and (b) are cross-sectional plots in one-dimensional form. (a) A stable spiral traveling wave for *b* = 1.05 and (b) an oscillatory spiral wave pattern for *b* = 1.025. (c) The alternans or oscillation of different spiral pulses for *b* = 1.032 as the development of time. The parameter values used in the simulation are taken from Table 1.

In this subsection, we also attempt to find a stable and oscillatory 1D parallel pulse train solution, which is analogous to the 2D spiral wave. Spiral waves are obviously two-dimensional, even so, they become one-dimensional as one moves away from the core so that the curvature decreases. We have described the dynamical behavior of spiral wave in the previous subsection. In this subsection, we show the dynamical behavior as time evolves.  Figure 7(a) represents the stable periodic spiral traveling wave solutions for *b* = 1.05 as a function of time, that is, before the bifurcation. Specifically, it shows the dynamical behavior of the spiral pulse propagation from the core to the boundary of the medium. The interpolation determines the values excluding one pulse near the spiral tip to avoid the complications dealing with trajectories in the vicinity of the spiral wave core. The width of every spiral pulse is equal in this case (i.e., 12.2628 for all pulses). The spiral pulses are not oscillating for *b* = 1.05.  Figure 7(b) illustrates the oscillatory pattern of wave propagation after the bifurcation, that is, for *b* = 1.025 and using the same computational settings.  Figure 7(c) shows the alternans or oscillation of spiral pulses as time develops. We avoided two pulses near the core and one pulse near the boundary during, since the spiral wave is symmetric on either side of the core, as we mentioned earlier.

#### 3.4.3. Corresponding Periodic Traveling Wave Solutions in 1D

**Figure 8:**
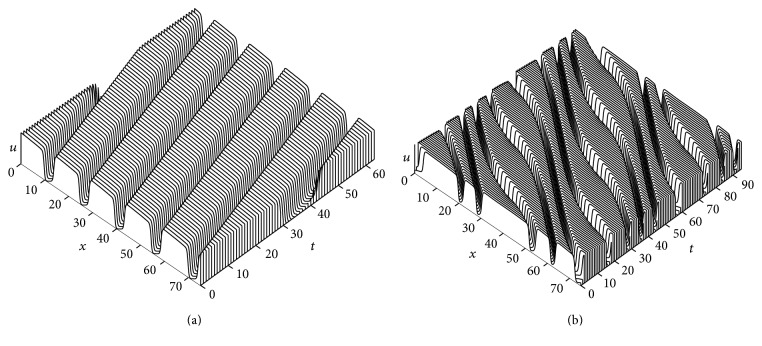
Space-time plots of integrations of (1) in one dimension. Time sequence illustrating the dynamics of (a) a stable periodic traveling wave solution for *b* = 1.05 and (b) an oscillatory pattern of solution for *b* = 1.025 in one dimension. The parameter settings are same as those in Table 1.

In this subsection, we attempt to make a comparison between one- and two-dimensional numerical results as a function of the parameter *b*. The computational settings are same as in Figure 7. For the numerical computation in one dimension, we used an implicit scheme with periodic boundary conditions on [0, *L*]. The system size of the medium is *L* = 75 with five pulses; that is, the spatial period is *l* = 15.  Figure 8(a) shows periodic traveling wave (PTW) solutions for *b* = 1.05, that is, before the bifurcation. We observed a stable wave pattern for the parameter *b* = 1.05.  Figure 8(a) corresponds to Figure 7(a). One can easily understand from these two Figures that the wave propagation speed and the pulse widths are equal in both cases.  Figure 8(b) illustrates the oscillatory pattern of wave propagation after the bifurcation, that is, for *b* = 1.025, in one dimension.  Figure 8(b) corresponds to Figure 7(b). The oscillatory patterns of wave and wave speed are also close in both cases. These results prove the dynamical correspondence or resemblance between the one-dimensional periodic traveling wave solutions (PTWs) and the spiral wave solutions. Therefore, the numerical result in two dimensions is a consequence of the result in one dimension.

### 3.5. Numerical Results for Vanishing Diffusion of the Recovery Variable

In the previous sections, we chose a small diffusion coefficient for the second component, since it is more advantageous to assume diffusion coefficient vanishingly small instead of zero for analytical treatment of the problem. In this case, the structure of the first order traveling wave ODE system is more regular. However, in the case of neuron or cardiac cell dynamics the recovery component does not diffuse. In this case, the model (1) usually has the following form:

(5) $$\begin{array}{c} \frac{\partial u}{\partial t}=d_{u}\Delta u+u\left(1-u\right)\left(u-a\right)-v,\\ \frac{\partial v}{\partial t}=\epsilon \left(du\left(b-u\right)\left(u+c\right)-v\right). \end{array}$$

**Figure 9:**
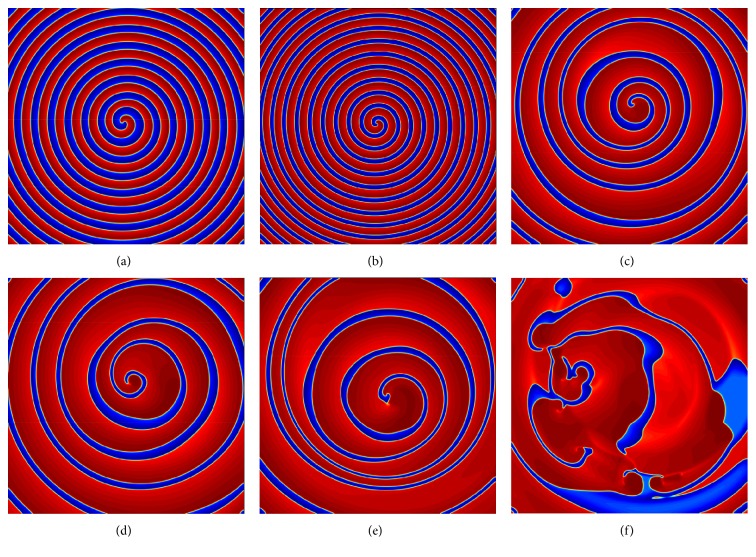
The spiral wave dynamics of the tissue as a function of the parameter *b* in (5). The other parameter values are in Table 1. The panels are at (a) *b* = 1.2, (b) *b* = 1.1, (c) *b* = 1.05, (d) *b* = 1.04, (e) *b* = 1.03, and (f) *b* = 1.02. Numerical integration with space step *dx* = *dy* = 0.25 and time step *dt* = 0.05 on the grid of 960 × 960 elements.

Figure 9 shows the dynamics of the spiral wave as a function of the parameter *b* of (5). Similarly, as before, we used the ADI method with Neumann boundary conditions. We continued the simulation for a long time for every value of *b* in order to arrive at a steady state of the solution. Figures 9(a) and 9(b) show a stable spiral pattern with an increased excited area as *b* decreases. The thickness of the spiral arm indicates the amount of depolarized tissue between the action potential upstroke and the depolarization phase. The initiation of oscillatory wave motion is presented in Figure 9(c), for *b* = 1.05. The oscillatory pattern progressively increases when *b* is further decreased as shown in Figures 9(d) and 9(e). On the spiral wave arm, the pulses are thinner in some areas and thicker in others. It is observed that increasing oscillations in wavelength or excited state on the spiral wave arm eventually leads to spiral breakup and formation of daughter spiral core [46]. This type of spiral breakup is known as far-field breakup [47]. Finally, as time evolves, breakup spreads across the entire medium (see Figure 9(f)).

**Figure 10:**
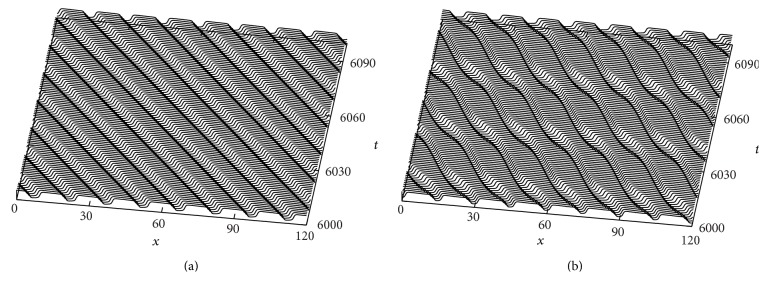
Space-time plots of integrations of (5) in one dimension. Time sequence illustrating the dynamics of (a) a stable PTW solution for *b* = 1.055 and (b) an oscillatory pattern of solution for *b* = 1.03. The parameter settings are same as in Figure 9.

Figure 10 shows a stable and an oscillatory pattern of PTWs in one dimension as a consequence of Figure 9. The parameter settings are the same as in Figure 9.  Figure 10(a) displays a stable wave pattern of the PTWs (or wave trains) for *b* = 1.055. In Figure 9, we observed that the onset of oscillation is close to *b* = 1.05 in two dimensions. As we gradually decrease the parameter *b*, it is noticed that the stable PTW solution bifurcates to an oscillatory wave pattern (see Figure 10(b)). However, the onset of oscillation is about *b* = 1.042. Figure 10(b) (at *b* = 1.03) corresponds to Figure 9(e). The oscillatory pattern of the wave and the wave speed are also consistent in both cases. These numerical observations indicate the dynamical resemblance between the one-dimensional PTWs and the spiral wave dynamics.

**Figure 11:**
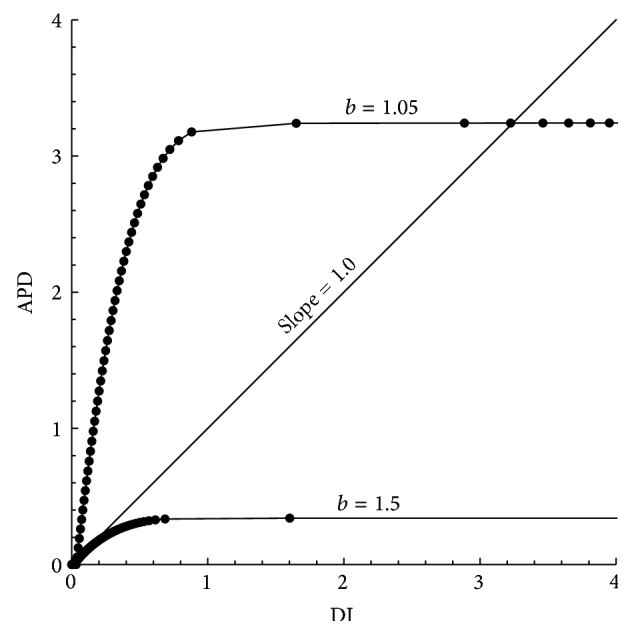
The APD restitution curves in model (5). The first one is for *b* = 1.5, having slope <1, and the second one is for *b* = 1.05, having slope > 1. The parameter settings are the same as in Figure 9.

In Figure 11, we computed the APD restitution curves for two different values of the parameter *b*. Usually, the restitution curve is defined as APD = *f*(DI), where APD indicates the duration of the action potential created by the second stimulus after a specific DI. As mentioned earlier, DI is the time interval between the end of the first action potential and the start of the second stimulus. Here, we simply desire to find a steeper restitution curve of (5) when the value of the parameter *b* decreases. The slope of the restitution curve can be related to the instability of the PTWs or spiral instability [40, 46]. Our results indicate that the smaller the *b*, the steeper the restitution curve (slope > 1) (see Figure 11). The slope of the restitution curve for small DI is defined as the ratio between the velocities on the left and the right branches at the level of the local peak of the cubic function. Since we also found spiral instability in the simulation results, at smaller values of *b*, the steepness of the APD restitution curve has a significant role in the process of spiral wave instability [34, 35].

## 4. Conclusions

We have studied the different phenomenon of spiral wave solutions in an excitable FHN-type of reaction-diffusion system. We observed in our numerical results that the process of spiral breakup is a consequence of the transverse instability of the planar traveling wave solutions. It was clearly demonstrated through numerical simulations that the parameter, *b*, is responsible for creating most of the characteristic properties of the cardiac excitable tissue. It plays a significant role in the demonstration of the dynamics of the spiral waves. As we mentioned earlier, the parameter, *b*, controls the gaps between the two nullclines on the right side of the *uv*-plane (see Figure 1(a)). Previous efforts (e.g., [16, 22, 28, 33]) did not consider the gaps between the two nullclines as a control parameter in order to study spiral wave dynamics. Our results showed that, by controlling the gaps, stable and unstable spiral patterns are generated in the medium. The spiral breakup occurs when the two nullclines are sufficiently near to each other. We also observed that a small decrease in *b* increases the pulse width or excited state. Our numerical results show that the stable spiral pattern bifurcates to an oscillatory pattern when *b* decreases from a stable wave pattern. However, in a specific parameter regime far below the stable pattern, the spiral wave becomes unstable and finally breaks up. We found that the spiral breakup is preceded by the alternans instability of spiral pulses far below the bifurcation. These results also suggest that spiral breakup can be controlled by reducing the excited state of the medium. Our model predicts that it is possible to arrive at a normal heart wave from the arrhythmias like alternans by a small reduction of excited area of the tissue. Thus, our model and simulation results can serve as a paradigm for further investigation of the cardiac arrhythmias in a rigorous mathematical treatment.

## Figures and Tables

**Algorithm 1:**
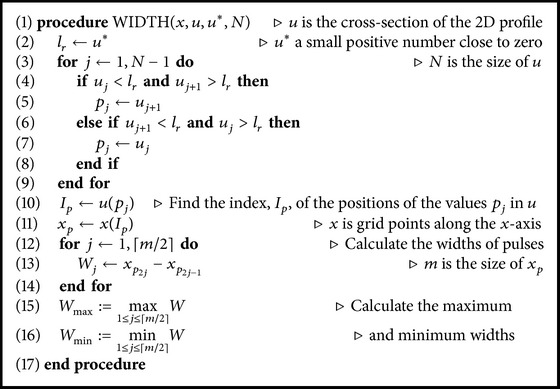
Calculate widths of different spiral pulses.

**Table 1:** Typical set of parameter values in (1) used in the numerical simulation.

Parameters	*a*	*b*	*c*	*d*	*d*_*u*_	*d*_*v*_	*ϵ*
Values	7 × 10^−2^	⋯	3.0	2.1 × 10^−1^	5 × 10^−2^	5 × 10^−3^	1.1 × 10^−2^
